# Cognition and attitudes of hospice care among healthcare providers: a case study of Sichuan Province

**DOI:** 10.1186/s12909-023-04898-7

**Published:** 2023-12-13

**Authors:** Meng Ling, Pengru Chen, Qiaoying He, Yi Long, Lei Cheng, Chuan You

**Affiliations:** 1 Department of Hepatobiliary Surgery, Affiliated Hospital of North Sichuan Medical College, No.1 Maoyuan North Road, Shunqing District, Nanchong, Sichuan China; 2 Health Management Center, Second Affiliated Hospital of North Sichuan Medical College, Nanchong, Sichuan China; 3 Nursing Teaching and Research Office, Nanchong Health School of Sichuan Province, Nanchong, Sichuan China; 4 College of Basic Medicine, Zunyi Medical University, Zunyi, Guizhou China

**Keywords:** Hospice care, Cognition, Attitudes, Hospital training

## Abstract

**Background:**

Under the background of the increasing aging population and cancer burden in China, the role of hospice care has become increasingly prominent. The government has paid more attention to the development of hospice care and set up pilot hospitals to promote hospice care. Moreover, healthcare providers play a leading role in hospice care services. To improve the quality of hospice care, the National Health Commission of the People's Republic of China proposed to set up hospice care training bases in municipal or above-level hospitals with hospice care or relevant work foundations, and train healthcare providers on hospice care. This study aimed to investigate the current situation of cognition and attitudes about hospice care among healthcare providers and provide a theoretical basis for hospital training.

**Methods:**

We used a quantitative design. A questionnaire survey was conducted among 1591 healthcare providers from August 2022 to November 2022. SPSS 22.0 software was used to analyze the data.

**Results:**

As a significant way of continuing education for healthcare providers, hospital training hasn't been effectively exploited in hospice care education. The average score of hospice care knowledge among participants was (7.74 ± 2.242) and the average score of hospice care attitudes among participants was (4.55 ± 1.503). According to multivariate linear regression analysis, sex (*p* < 0.001), education levels (*p* < 0.001), and professional titles (*p* = 0.018) of participants had significant difference on the score of hospice care knowledge; education levels (*p* = 0.009) and professional titles (*p* = 0.016) of participants had significant difference on the score of hospice care attitudes.

**Conclusions:**

There were some misunderstandings about hospice care among healthcare providers and their attitudes towards hospice care were inactive. It's suggested that hospitals should carry out professional and systematic education courses to help healthcare providers understand hospice care correctly, and participate in hospice care services actively.

## Introduction

Hospice care is a special type of care provided by hospice team members, including doctors, nurses, home health aides, social workers, clergymen or other counselors, trained volunteers, etc., who are committed to supporting the medical, psychological, and spiritual needs of both patients (i.e., the life expectancy of 6 months or less) and their loved ones [1]. The role of hospice care in relieving the pain, suffering of patients with advanced diseases and improving their quality of life has been widely recognized [2]. Especially under the social background of increasing cancer burden [3], and deepening population aging [4] which was also accompanied by family miniaturization and empty nest [4, 5], the role of hospice care is increasingly prominent [6].

Systematic hospice services originated in the UK, marked by St. Christopher's Hospice which was founded by Dr. Cicely Saunders in 1967. During the last decades, the development of hospice care services has increased gradually. Home care teams, hospices, support teams in hospitals, and specialized hospice care units have been established in most countries [7]. In the UK, among the general public, there was a high preference for hospice care. Home hospice care is the most common preference, with inpatient hospice care as the second preference in advanced illness [8]. Healthcare providers, especially general practitioners (GPs), play an important role in hospice care [9–11].

Education and training in hospice care are highly valued in other countries. Hospice has now been recognized by the American Board of Medical Subspecialties as a field with a unique body of knowledge and practice. With 9 other specialty boards, the American Board of Emergency Medicine has cosponsored hospice and palliative medicine as an official subspecialty [12]. In Germany, nursing staff can undergo additional training in hospice care. By doing so, they can offer patients different kinds of nursing care that cater more to their mental, social, and spiritual welfare than primarily to matters of hygiene or other physical needs [13]. The Japanese Society for Palliative Medicine developed the "Palliative care Emphasis program on symptom management and Assessment for Continuous medical Education" (PEACE) in 2008. PEACE was designed to teach symptom management and communication skills, etc. to all healthcare providers who engage in cancer care [14, 15]. The development of PEACE promoted a significant improvement in basic hospice care education for physicians engaged in providing cancer care [16].

Modern hospice care in China began in the twentieth century. In 1982, hospice service was launched by Notre Dame in Kowloon, Hong Kong [17]. In 1983, Professor Keshi Zhao began to promote the home care program for patients with terminal cancer in Taiwan [18]. In 1988, the Hospice Research Center of Tianjin Medical University was established, which opened the prelude to hospice care in mainland China [19]. In 1990, the Beijing Songtang Care Hospital, Shanghai Nanhui Care Hospital [20] and Taiwan Mackay Memorial Hospital began providing hospice care service [21], then Xi'an, Shenyang, Guangzhou, and other cities followed successively [20]. In 1998, Li Ka Shing Foundation cooperated with 20 hospitals across our country to provide home nursing services for advanced cancer patients free of charge [19].

In recent years, China has paid more attention to the development of hospice care. In October 2016, "Outline of Healthy China 2030 Plan" clearly stated:" to provide elderly persons with treatment hospitalization, rehabilitation care, stable life care, and hospice care integration for health and pension services [22]." In December of the same year, "China's 13th Five-Year Plan for Health and Wellness" proposed to develop and strengthen long-term care, chronic disease management, hospice care, and other continuing medical institutions [23]. Then, the "Hospice Care Practice Guidelines (Trial)" "Basic Standards and Management Norms for Hospice Care Centers (Trial)" and other documents were issued in 2017, which put forward requirements for the number of beds, department settings, staffing, etc. of hospice care centers, and standardized the symptom control, psychological and social support services, etc. of hospice care. Furthermore, the first batch of hospice care pilots was launched in the Haidian District of Beijing, Changchun City of Jilin Province, Putuo District of Shanghai, Luoyang City of Henan Province, and Deyang City of Sichuan Province [24]. After that, hospice care projects were included in the scope of medical insurance reimbursement in our country [25], which helps reduce the economic burden of dying patients and their families. In 2019, the National Health Commission of the People's Republic of China announced 71 cities/districts, including Beijing and Shanghai, as the second batch of hospice care pilot units [26]. By the end of 2020, there were 510 hospitals with hospice departments in China [24].

To improve the quality of hospice care, the National Health Commission of the People's Republic of China proposed to set up hospice care education and training bases in municipal or above-level hospitals which with relevant work foundations, and train healthcare providers on hospice care conception and skills, etc. [26]. Therefore, we selected healthcare providers who worked in tertiary hospitals as the research objects, and investigated their cognition and attitudes about hospice care through a questionnaire survey, to provide reference for setting up hospice training base and carrying out hospice care education in hospitals.

 This study reports an analysis of quantitative data and answers to the following research questions:

1. What is the knowledge and attitudes of healthcare providers about hospice care?
2. What factors help us to understand the variation in knowledge and attitudes?

## Methods

This study used a quantitative, descriptive design. The approach enabled the data collection and analysis objectively. Ethical approval was received from the Affiliated Hospital of North Sichuan Medical College.

### Study design

Referring to the Frommelt Attitude Toward Care of the Dying Scale (FATCOD) in America [27, 28] and previous research [20, 29] in China, we designed a questionnaire to investigate the cognition and attitudes of healthcare providers towards hospice care. The scale of the questionnaire consists of 18 questions, and exploratory factor analysis extracted two common factors. One of the questions (Q1) has a load higher than 0.5 in the two factors, it is invalid and deleted. The remaining 17 questions are valid. Besides, based on the results of exploratory factor analysis, the first factor contains 10 questions and the second factor contains 7 questions. Moreover, according to the previous research [20, 27–29] and the content of each question, the first factor (10 questions) is determined as cognition of hospice care, and the second factor (7 questions) is the attitudes toward hospice care. The cumulative variance contribution rate was 55.484%.

After expert consultation and preliminary investigation, the questionnaire was determined. The Cronbach's α coefficient of the total scale was 0.933, the split-half reliability of the scale was 0.912, and the Cronbach's α coefficients of two dimensions were 0.914, and 0.810, respectively. In addition, the KMO=0.941, Barlette test was used (*p*<0.001). The study conforms to the ethical requirements.

### Participants

We selected five tertiary hospitals with hospice care or related medical services in Sichuan Province in China. On the one hand, hospice care services have been existing in Sichuan Province since 1998 [19]. On the other hand, based on the hospital management plan proposed by the Ministry of Health, hospitals are divided into three levels in China. The tertiary hospitals stand at the highest level and integrate clinical, teaching, and scientific research. They assume the responsibility of providing guidance and training to lower-level hospitals. So, as research subjects, they are more representative than other levels of hospitals.

The inclusion criteria for this study were formal healthcare providers who work in the chosen hospitals and get health professional qualifications. Exclusion criteria included: those without health professional qualifications, and interns. Eligible participants were approached and consented virtually.

### Data collection and measures

Our study was conducted from August 2022 to November 2022, the survey was primarily performed in the inpatient ward of hospice care, cardiology, geriatrics, oncology, gastroenterology, intensive care unit, and respiratory in the selected hospitals. Healthcare providers were asked by trained interviewers whether they would like to participate in the survey.

Our questionnaires have three parts: demographic information, knowledge about hospice care, and attitudes towards hospice care. In the first part, we collected the participants' backgrounds including sex, age, religion, education levels, occupation, professional titles, etc. In the second part, we assessed the level of knowledge among participants by ten objective questions (0–1 point per question out of 10). In the third part, seven questions (0–1 point per question out of 7) were designed to assess participants' attitudes towards hospice care. Each questionnaire declared that participation in the study is voluntary, and the responses would be only used for research. To assure confidentiality, the instructions also stated that completed survey questionnaires would not be revealed to anyone other than the researchers.

### Data analysis

All data were analyzed by Statistical Package for the Social Sciences (SPSS, version 22.0). Descriptive statistics were calculated for all study variables. Associations of general characteristics with cognition and attitudes on hospice care were tested by t-test and one-way ANOVA. Predictors of healthcare providers' cognition and attitudes towards hospice care were examined through multiple regression techniques. Pearson correlation analysis was applied for the correlation between cognition and attitudes. For all analyses, *p* < 0.05 was considered statistically significant.

## Results

### General characteristics of study participants

A total of 1600 individuals participated in the questionnaire filling in our survey, with a response rate of 99.4%. Due to some external or uncontrollable factors, 9 participants failed to complete the questionnaire. Sociodemographic characteristics are presented in Table 1. The majority of participants were female (*n* = 845, 53.1%), and the mean age was (34.24 ± 9.19) years old (range 19–55). Most of the participants only got bachelor degree (*n* = 843, 53.0%). There were 998 (62.7%) doctors and 593 (37.3%) nurses, most of them with the primary title (*n* = 923, 58.0%). Approximately 92.6% (*n* = 1473) of the participants have heard of hospice care, and 62.5% (*n* = 994) of these participants learn about hospice care through school education.

**Table 1:** General characteristics of the participants (*N* = 1591)

Variables	N (%)
Sex	
Male	746 (46.9)
Female	845 (53.1)
Age	
≤ 25	318 (20.0)
26 ~ 35	623 (39.2)
36 ~ 45	373 (23.4)
≥ 46	277 (17.4)
Marital status	
Married	1142 (71.8)
Unmarried	449 (28.2)
Religion	
Yes	68 (4.3)
No	1523 (95.7)
Educational levels	
Associate Degree	478 (30.0)
Undergraduate	843 (53.0)
Postgraduate	270 (17.0)
Occupation	
Doctor	998 (62.7)
Nurse	593 (37.3)
Professional titles	
Primary title	923 (58.0)
Intermediate title	346 (21.8)
Senior title	322 (20.2)
Have heard about hospice care	
Yes	1473 (92.6)
No	118 (7.4)
Hospice knowledge sources	
School education	994 (62.5)
Media or internet	780 (49.0)
Hospital training	706 (44.4)
Others	370 (23.3)

### Knowledge about hospice care

Knowledge about hospice care among the participants is shown in Table 2. The average score of knowledge among participants was (7.74 ± 2.242), and the total mark was 10 points. Referring to the evaluation standard: of 70 points out of 100 [29], the participants scored a little high on hospice care knowledge but still with some misunderstanding of hospice care. Approximately, 91.2% (*n* = 1451) of the participants believed that hospice care could improve the quality of life for terminal patients, and 67.3% (*n* = 1072) of the participants considered that hospice care could be utilized not only for patients with advanced cancer. Most of the participants' supposed hospice care needs interdisciplinary teamwork (*n* = 1416, 89.0%) and focuses on care (*n* = 1155, 72.6%), neither lengthens nor shortens one's survival time (*n* = 1237, 77.7%). However, 7.7% (*n* = 123) of the participants still regarded hospice care as euthanasia (Table 2).

**Table 2:** The score of knowledge and attitudes about hospice care among the participants (*N* = 1591)

Question	Yes (%)	No (%)	Not Sure (%)
**Knowledge**			
1. In hospice care service, treatment is more important than care. (No)	260 (16.3)	1155 (72.6)	176 (11.1)
2. Terminal patients with a definite diagnosis, hopeless treatment, and expected life within 6 months belong to the scope of hospice care. (Yes)	1072 (67.3)	227 (14.3)	292 (18.4)
3. Hospice care service is provided by a team composed of doctors, nurses, social workers, volunteers, etc. (Yes)	1416 (89.0)	63 (4.0)	112 (7.0)
4. Hospice care has many modes include hospital hospice, hospice wards, community hospice or home hospice, etc. to meet the different needs of patients and their families. (Yes)	1408 (88.5)	69 (4.3)	114 (7.2)
5. Hospice care provides palliative or supportive medical measures, neither delaying nor accelerating the death. (Yes)	1237 (77.7)	163 (10.2)	191 (12.1)
6. Hospice care can improve the quality of life of dying people and make them leave peacefully, which is consistent with the maintenance of human dignity too. (Yes)	1451 (91.2)	64 (4.0)	76 (4.8)
7. Hospice care is euthanasia. (No)	123 (7.7)	1298 (81.6)	170 (10.7)
8. Pain control is one of the most important aspects of hospice care.(Yes)	1300 (81.7)	133 (8.4)	158 (9.9)
9. 80% ~ 90% of the pain can be controlled as long as the drug selection is appropriate, the dosage is reasonable, and the interval of administration is proper. (Yes)	982 (61.7)	266 (16.7)	343 (21.6)
10. The clients of hospice care include not only the hospice patients but also their families, which should last until the mourning period. (Yes)	1011 (63.5)	324 (20.4)	256 (16.1)
**Attitudes**			
1. Healthcare providers pay more attention to the general patient than to the dying patients and ignore the needs of the dying. (No)	546 (34.3)	735 (46.2)	310 (19.5)
2. Hospice care lacks social support in China. (Yes)	1081 (67.9)	178 (11.2)	332 (20.9)
3. Hospice care against the Chinese tradition of filial piety. (NO)	100 (6.3)	1333 (83.8)	158 (9.9)
4. Hospice care goes against the traditional medical concept of "healing the wounded and rescuing the dying". (No)	133 (8.4)	1333 (83.8)	125 (7.9)
5. Hospice care is conducive to relieving the financial and mental pressure of the dying and their families. (Yes)	1171 (73.6)	221 (13.9)	199 (12.5)
6. Hospice care is beneficial to the rational allocation of medical resources. (Yes)	1238 (77.8)	134 (8.4)	219 (13.8)
7. Caring for the dying is increasing the psychological burden of healthcare providers inevitably. (No)	875 (55.0)	351 (22.1)	365 (22.9)

The score of hospice care knowledge among health care providers was significantly associated with sex (t = 2.994, *p* = 0.003), age (F = 5.698, *p* = 0.001), education levels (F = 14.275, *p* < 0.001), and professional titles (F = 16.023, *p* < 0.001). The score of hospice care knowledge was significantly higher in female and older respondents; in health care providers got postgraduate degree and senior title (Table 3).

**Table 3:** Analysis of knowledge and attitudes about hospice care among the participants (*N* = 1591)

**Variables**	**Knowledge score** ($\overline{X } \pm S$)	***F/t***	***p***	**Attitudes score** ($\overline{X } \pm S$)	***F/t***	***p***
Sex		2.994	0.003		1.235	0.217
Male	7.56 ± 2.425			4.50 ± 1.511		
Female	7.90 ± 2.055			4.59 ± 1.458		
Age		5.698	0.001		5.378	0.001
≤ 25	7.65 ± 2.152			4.35 ± 1.545		
26 ~ 35	7.55 ± 2.400			4.48 ± 1.552		
36 ~ 45	7.82 ± 2.357			4.66 ± 1.474		
≥ 46	8.19 ± 1.689			4.79 ± 1.336		
Marital status		-1.402	0.161		-2.488	0.013
Unmarried	7.62 ± 2.295			4.39 ± 1.592		
Married	7.79 ± 2.219			4.61 ± 1.462		
Religion		1.806	0.071		-0.232	0.816
Yes	7.26 ± 2.290			4.59 ± 1.406		
No	7.77 ± 2.238			4.54 ± 1.507		
Educational levels		14.275	< 0.001		3.933	0.020
Junior College	7.36 ± 2.309			4.40 ± 1.546		
Undergraduate	7.81 ± 2.187			4.58 ± 1.464		
Postgraduate	8.24 ± 2.179			4.70 ± 1.526		
Occupation		-1.468	0.142		-0.301	0.763
Doctor	7.68 ± 2.346			4.54 ± 1.534		
Nurse	7.85 ± 2.053			4.56 ± 1.450		
Professional titles		16.023	< 0.001		12. 473	< 0.001
Primary title	7.53 ± 2.308			4.42 ± 1.542		
Intermediate title	7.75 ± 2.342			4.55 ± 1.478		
Senior title	8.35 ± 1.790			4.90 ± 1.353		

### Attitudes towards hospice care

Attitudes towards hospice care among the participants are presented in Table 2. The average score of attitudes among participants was (4.55 ± 1.503), and the total mark was 7 points. Referring to the evaluation standard: of 70 points out of 100 [19], the attitudes of the participants towards hospice care were inactive. Most of the participants (*n* = 1333; 83.8%) considered that hospice care conforms to traditional medical ideas. A total of 77.8% (*n* = 1238) answered "Yes" regarding the importance of hospice care to promote optimal allocation of medical resources, and 73.6% (*n* = 1171) believed in hospice care could relieve families' financial pressure. However, 55.0% (*n* = 875) of the respondents were reluctant to participate in hospice services because they insisted that caring for dying patients would increase their psychological burden.

Attitudes towards hospice among the participants were significantly associated with age (F = 5.378, *p* = 0.001), marital status (t = -2.488, *p* = 0.013), education levels (F = 3.933, *p* = 0.020), and professional titles (F = 12.473, *p* < 0.001). Hospice care attitudes were significantly more positive among healthcare providers who were married and aged over 46 years old; in those who got postgraduate degree and senior title (Table 3).

### Multiple regression analysis of influencing factors about hospice care knowledge and attitudes among the participants

According to the generalized multiple linear regression model, it's assumed that there were $k$ independent variables of $\widehat{Y}$ related to the dependent variables (the score of hospice care knowledge and attitudes), which were marked as ${X}_{1}$, ${X}_{2}$, …, ${X}_{k}$ and the multiple linear regression equation was assumed to be:

$$\widehat{Y}= {b}_{0} + {b}_{1}{X}_{1} + {b}_{2}{X}_{2} +... + {b}_{k}{X}_{k} +e$$

According to the results of the t-test and one-way ANOVA, the score of knowledge was used as the dependent variable, and sex, age, education levels, and professional titles were used as the independent variables. Besides, the score of attitudes was taken as the dependent variable, and marital status, age, education levels, and professional titles were used as independent variables (Table 4).

**Table 4:** Variable assignment

Variable	Server variable	Assignment description
Dependent Variable		
Knowledge score		**Continuous variable**
Attitudes score		**Continuous variable**
Independent variable		
Sex	*X*_1_	Male = 1, Female = 0
Age	*X*_2_	≤ 25 = 000, 26 ~ 35 = 100, 36 ~ 45 = 010, ≥ 46 = 001
Marital status	*X*_3_	Unmarried = 0, Married = 1
Education levels	*X*_5_	Junior College = 00, Undergraduate = 10, Postgraduate = 01
Professional titles	*X*_6_	Primary title = 00, Intermediate title = 10, Senior title = 01

We conducted multiple regression analyses to examine factors influencing knowledge about hospice care among the participants. Sex, age, education levels, and professional titles were entered in the regression model as predictors of knowledge score about hospice care. Factors significantly predicted knowledge about hospice care, including the provision of sex (*p* < 0.001), education levels (*p* < 0.001), and professional titles (*p* = 0.018) (Table 5). *R*^2^ = 0.43, Adjusted *R*^2^ = 0.41. The regression equation was:

**Table 5:** Regression analyses of influencing factors of knowledge score

Independent variable	*B*	*SE*	*β*	*t*	*p*	95%* CI*
**Constant**	7.481	0.141		53.058	< 0.001	7.204 ~ 7.757
Sex	-0.519	0.116	-0.116	-4.466	< 0.001	-0.747 ~ -0.291
Age	0.247	0.249	0.042	0.989	0.323	-0.242 ~ 0.735
Education levels	0.937	0.179	0.157	5.224	< 0.001	0.585 ~ 1.289
Professional titles	0.510	0.216	0.091	2.361	0.018	0.086 ~ 0.934
	***R***^***2***^				0.43	
	***Adjusted R***^***2***^				0.41	
	***F***				9.770	
	***P***				< 0.001	

$$\mathrm{Knowledge\,score }=7.481-0.519 \times \mathrm{Sex\,}+0.937 \times\,\mathrm{Education\,levels }+0.510 \times \mathrm{Professional\,titles}$$  

Multiple regression analyses also were conducted to examine factors influencing attitudes about hospice care among the participants. Marital status, age, education levels, and professional titles were entered in the regression model as predictors of attitudes score about hospice care. Factors significantly predicted attitudes about hospice care, including the provision of education levels (*p* = 0.009) and professional titles (*p* = 0.016) (Table 6). *R*^2^ = 0.17, Adjusted *R*^2^ = 0.15. The regression equation was:

**Table 6:** Regression analyses of influencing factors of attitudes score

Independent variable	*B*	*SE*	*β*	*t*	*p*	95%* CI*
**Constant**	4.265	0.095		44.896	< 0.001	4.079 ~ 4.452
Age	0.143	0.198	0.036	0.725	0.469	-0.244 ~ 0.531
Marital status	0.004	0.126	0.001	0.032	0.975	-0.243 ~ 0.251
Education levels	0.300	0.114	0.075	2.631	0.009	0.076 ~ 0.524
Professional titles	0.356	0.147	0.095	2.421	0.016	0.068 ~ 0.645
	***R***^***2***^				0.17	
	***Adjusted R***^***2***^				0.15	
	***F***				3.779	
	***P***				< 0.001	

$$\mathrm{Attitudes\,score }=4.265\,+0.300\,\times\,\mathrm{Education\,levels }+0.356\,\times\,\mathrm{Professional\,titles}$$  

### Correlation between knowledge and attitudes of hospice care

The knowledge of hospice care was positively correlated with the attitudes towards hospice care among healthcare providers (*r* = 0.544, *p* < 0.001). Cognition determined attitudes. The higher score of hospice knowledge was, the more positive hospice attitudes were.

## Discussion

In this study, the knowledge score of healthcare providers towards hospice care reached the passing standard, but, some items also needs to be enriched: Pain control, scope of care, service recipients. First of all, the three-step analgesia principle which instructed by WHO was adopted in hospice care. Combined with pain assessment scales or tools, the pain can be effectively controlled [30, 31]. In addition, some participants consider that hospice care was only for patients with advanced cancer. Actually, there was no limited by disease type in hospice care. Hospice care is available to all patients whose estimated survival time is less than six months and have willing to accept the care. Moreover, the patient's families also need caring [1].

Sex had a statistically significant impact on the score of health care providers among hospice care knowledge. Women scored higher, which was different from previous studies [32]. As we all know, hospice care services require healthcare providers not only to master professional knowledge and skill but also to have the ability to empathize with dying patients and their families [33–35]. From the perspective of caring ethics, men prefer to look for answers logically. Nevertheless, women tend to put themselves in the person's shoes and consider the physiological and psychological feelings of others concerned [36]. Thus, women may get the meaning of hospice care better. It suggests that hospitals should emphasize the primary position of patients and their families, and pay more attention to cultivating the communication skills of health care providers, then help them learn how to make decisions from the perspective of patients and their families. The SPIKES mode [37–39] and SHARE mode [40–42] are available for reference.

The participants with higher education levels and professional titles also scored higher on hospice care knowledge and attitudes, but there were still misconceptions about hospice care among healthcare providers [43]. According to relevant policies, professional title promotions of healthcare providers have education levels and working seniority requirements. Under normal circumstances, those with higher professional titles also with higher education levels and richer clinical working experiences. In a great measure, their cognition of hospice care may come from teaching/training and years of clinical experiences. On the one hand, we did find that most healthcare providers obtained hospice care knowledge through school education (*n* = 994, 62.5%). However, hospice care courses were offered in some universities, and it's not sufficient even in medical schools in China [44]. Besides, the contents about hospice care only appeared in elective courses or one chapter of specialized courses [45–47]. Most undergraduate students had difficulty recalling what they had been taught about hospice care [47], not to mention healthcare providers [43]. On the other hand, based on their years of clinical experiences, healthcare providers could find the answer to the questions about hospice care. However, due to the lack of systematic training, healthcare providers can’t realize the necessity and value of hospice care [48, 49]. Healthcare providers believed they would benefit from further hospice care training, especially in symptom management and opioid titration [50, 51]. And, as an important way of hospice care education for healthcare providers, hospital training [7, 52] hasn't been effectively exploited in hospice care, only accounting for 44.4% (*n* = 706).

Furthermore, because of the misunderstanding of hospice care, even among healthcare providers who know about the value of hospice care, they may still equate hospice care to "giving up" or agreeing to shorten one's life [53] and insist that hospice care violates the principle of lifesaving for terminal patients [19]. It is noteworthy that those who had formal hospice education or training had more knowledge and positive attitudes toward hospice care than those without such education or training [54, 55]. Hospice care training is beneficial to changing the opinion regarding hospice care ethics and utilization rate among healthcare providers [55, 56]. Therefore, it's suggested that hospitals should carry out hospice care training to make up for the lack of hospice care in school education. In consideration of there is less professional training on hospice care in China at present, it's recommended hospitals refer to successful experiences in other countries, such as the "Quality End of Life Care for All (QELCA)" training launched by St Christopher's Hospice in the UK [33], and the "End-of-life Nursing Education Consortium (ELNEC)" course from the USA [34]. As early as 2016, the Beijing Living Will Promotion Association (LWPA) began collaborating with St. Christopher's Hospice to promote the QELCA training in China [57]. The QELCA training emphasizes the combination of theoretical teaching and action learning. In the field of hospice care, the positive effects of QELCA training in developing the knowledge and skills of healthcare providers have been proven [58]. Moreover, it also enables healthcare providers, teams, and hospitals/organizations to make positive attitudes towards hospice care [59].

Influenced by traditional concepts, people always avoid talking about death in China. Death education was rarely found in mainland China [60]. Therefore, many healthcare providers still fear facing death [61]. This may be the reason why some healthcare providers (*n*=875, 55.0%) feel that providing hospice care services will increase their psychological pressure [49]. So, hospital training should include death education.

On the whole, sex, education levels, and professional titles had statistical significance on the score of hospice care knowledge; educational levels and professional titles had statistical significance on the attitudes towards hospice care in this research. However, the differences between these groups were small. It's worth emphasizing that hospice care education/training has a positive impact on the knowledge and attitudes of healthcare providers [55, 62, 63]. We think that the small differences may confirm the positive effects of hospice care education/training, and the statistically significant ones indicate deficiencies in hospice care education/training. In recent years, our government, hospitals, and other institutions have taken a series of measures to promote the development of hospice care, including education/training for healthcare providers [24, 26]. It's helpful to improve the cognition and attitudes of healthcare providers towards hospice care. But, as we mentioned in this research, there are still some deficiencies in the form or content of the existing hospice care education/training that need to be improved. Based on the results of the study, we make some recommendations. They have reference value for education/training, cultivating professional service teams, and conducting further research in hospice care.

This study investigated the cognition and attitudes about hospice care among healthcare providers, analyzed its influencing factors, and provided recommendations. However, the study also has some potential limitations that are worth considering. Firstly, the hospitals selected in our study are located in Sichuan Province, which may limit their generalizability to the broader healthcare providers in China. Secondly, there was a lack of practice or reports about the use of ELNEC courses in hospice care training in China, the role of this course remains to be verified. Thirdly, we have identified some influencing factors, but, other factors haven’t been addressed and the research needs to be further explored. Finally, limited by time and human resources, no intervention research has been implemented. In the next step, we plan to do some interventions and conduct qualitative research.

## Conclusions

From the perspective of cognition and attitudes towards hospice care among healthcare providers, we conducted a questionnaire survey. In our study, the healthcare providers with higher educational levels and professional titles also got a higher score on hospice care knowledge and attitudes. Women scored higher than men on hospice care knowledge. In general, there were some misconceptions and inactive attitudes about hospice care among healthcare providers.

Most of the participants got hospice care knowledge from school education. However, hospice care education isn't sufficient even in medical schools in China. Moreover, as a significant way of hospice care education for healthcare providers, hospital training hasn't been effectively exploited. Our study suggests that hospital training should make up for the lack of hospice care in school education, add more content about hospice care, such as quality of life, patients' rights, etc., and pay more attention to the needs of patients and their families. Finally, help patients to end well.

## Data Availability

The datasets used and analyzed during the current study are available from the corresponding authors upon a reasonable request.

## References

[1] National Cancer Institute at the National Institute of Health (USA). Choices for care when treatment may not be an option. https://www.cancer.gov/about-cancer/advanced-cancer/care-choices. 2022. Accessed 8 Apr 2022.

[2] Harding R, Higginson IJ (2005). Palliative care in sub-Saharan Africa. Lancet.

[3] Chen WQ, Zheng RS, Baade PD (2016). Cancer statistics in China, 2015. CA Cancer J Clin.

[4] National Bureau of Statistics of China. China Statistical Yearbook 2021. 2022. http://www.stats.gov.cn/tjsj/ndsj/2022/indexch.htm. Accessed 16 Feb 2023.

[5] National Health Commission of the People's Republic of China.Published online by the National Health and Family Planning Commission (May 15, 2015). 2015. http://www.nhc.gov.cn/xcs/zxfb/201505/2e92dede38164863a112da225e1dbaeb.shtml. Accessed 8 Apr 2022.

[6] Liu JT, Yuan M (2016). History, present situation, problems and prospect of hospice service system in mainland China. J Soc Work.

[7] Pype P, Stes A, Wens J (2012). The landscape of postgraduate education inpalliative care for general practitioners:results of a nationwide survey in Flanders. Belgium Patient Educ Couns.

[8] Higginson IJ, Sen-Gupta GJA (2000). Place of care in advanced cancer: a qualitative systematic literature review of patient preferences. J Palliat Med.

[9] Mitchell S, Loew J, Millington-Sanders C (2016). Providing end-of-life care in general practice: findings of a national GP questionnaire survey. Br J Gen Pract.

[10] Borgsteede S, Graafland-Riedstra C, Deliens L (2006). Good end-of-life care according to patients and their GPs. Br J Gen Pract.

[11] Abarshi E, Onwuteaka-Philipsen B, Donker G (2009). General practitioner awareness of preferred place of death and correlates of dying in a preferred place: a nationwide mortality follow-back study in the Netherlands. J Pain Symptom Manage.

[12] Quest TE, Marco CA, Derse AR (2009). Hospice and palliative medicine: new subspecialty, new opportunities. Ann Emerg Med.

[13] Walker A, Breitsameter C (2015). Ethical decision-making in hospice care. Nur Ethic.

[14] Tsuneto S (2013). Past, present, and future of palliative care in Japan. Jpn J Clin Oncol.

[15] Morita T, Kizawa Y (2013). Palliative care in Japan: a review focusing on care delivery system. Curr Opin Support Palliat Care.

[16] Maeda I, Tsuneto S, Miyashita M (2014). Progressive development and enhancement of palliative care services in Japan: nation wide surveys of designated cancer care hospitals for three consecutive years. J Pain Symptom Manag.

[17] Qiu SH (2015). A review on problems of China's hospice care and analysis of possible solutions. Chinese Med J (Engl).

[18] Shen LL, Liu BB, Zhao LM (2017). The development and inspiration of hospice care in Taiwan. J Nurs Adm.

[19] Li J, Davis MP, Gamier P (2011). Palliative medicine:barriers and developments in mainland China. Curr Oncol Rep.

[20] Li YT, Li W, Liu F (2015). Hospice care.

[21] Li YT, Luo JL (2018). Research on the construction of hospice care medical servicesystem.

[22] China Central Committee and the State Council. Outline of Healthy China 2030 Plan. http://www.gov.cn/xinwen/2016-10/25/content_5124174.htm. 2016. Accessed 8Apr 2022.

[23] State Council. China's 13th Five-Year Plan for Health and Wellness. http://www.gov.cn/zhengce/content/2017-01/10/content_5158488.htm. 2017. Accessed 8 Apr 2022.

[24] National Health Commission of People’s Republic of China. Letter on reply to proposal 0296 (Social Management Class 027) of the Fourth Session of the 13th National Committee of the Chinese People’s Political Consultative Conference. http://www.nhc.gov.cn/wjw/tia/202112/3d615d9baf444954903a905d5d85ece2.shtml. 2021. Accessed 8 Apr 2022.

[25] National Health Commission of People’s Republic of China. Letter on reply to proposal 3011 (Social Management Class 278) of the Fifth Session of the 12th National Committee of the Chinese People’s Political Consultative Conference. 2018 http://www.nhc.gov.cn/wjw/tia/201801/162783d72e26439ea4d0d670e76d79bf.shtml. Accessed 8 Apr 2022.

[26] General Office of the National Health Commission of People’s Republic ofChina. Notice on carrying out the second batch of hospice care pilot work. 2019. http://www.nhc.gov.cn/lljks/s7785/201912/efe3ed3d9dce4f519bc7bba7997b59d8.shtml. Accessed 8 Apr 2022.

[27] Frommelt KH (2003). Attitudes toward care of the terminally ill: an educational intervention. Am J Hosp Palliat Care.

[28] Frommelt KH (1991). The effects of death education on nurses' attitudes toward caring for terminally ill persons and their families. Am J Hosp Palliat Care.

[29] Zheng YP. Study on hospice care knowledge s attitude of physicians and nurses and relation factors. Changsha, Hunan Province, China: PhD Thesis, Central South University; 2008.

[30] Ludvigsson C, Isaksson U, Hajdarevic S (2020). Experiencing improved assessment and control of pain in end-of-life care when using the Abbey Pain Scale systematically. Nurs Open.

[31] Wilkie DJ, Yao YW, Ezenwa MO (2020). A stepped-wedge randomized controlled trial: effects of health interventions for pain control among adults with cancer in hospice. J Pain Symptom Manage.

[32] Liu DD. Hospice care situation investigation and related factors analysis in Meizhou. Guangzhou, Guangdong Province, China: Master's thesis, Jinan University; 2011.

[33] Gillett K, Bryan L (2016). 'Quality End of Life Care for All' (QELCA): the national rollout of an end-of-life workforce development initiative. BMJ Support Palliat Care.

[34] Sherman DW, Matzo LP, Pitorak E (2005). Preparation and care at the time of death: content of the ELNEC curriculum and teaching strategies. J Nurses Staff Dev.

[35] Randall TC, Wearn AM (2005). Receiving bad news: patients with haematological cancer reflect upon their experience. Palliat Med.

[36] Li DD (2015). Comparison between Western Caring Ethics and Confucian Benevolence Ethics. Academic Exchange.

[37] Baile WF, Buckman R, Lenzi R (2000). SPIKES-A six-step protocol for delivering bad news:application to the patient with cancer. Oncologist.

[38] Mirza RD, Ren M, Agarwal A (2019). Assessing patient perspectives on receiving bad news: a survey of 1337 patients with life-changing diagnoses. AJOB Empir Bioeth.

[39] Krieger T, Salm S, Dresen A (2022). Cancer patients' experiences and preferences when receiving bad news: a qualitative study. J Cancer Res Clin Oncol.

[40] Fujimori M, Uchitomi Y (2009). Preferences of cancer patients regarding communication of bad news: a systematic literature review. Jpn J Clin Oncol.

[41] Tang WR, Chen KY, Hsu SH (2014). Effectiveness of Japanese SHARE model in improving Taiwanese healthcare personnel's preference for cancer truth telling. Psychooncology.

[42] Wang SH, Li YL, Wang H (2020). Application of SHARE model in hospice care. Int J Nurs.

[43] Ning XH (2019). Hospice and palliative care research in mainland China: current status and future direction. Palliat Med.

[44] Zhang XF, Tang SY (2017). Research progress in hospice care education and training. Chin J Gerontol.

[45] Zhang J, Peng Y, Wang ZR (2010). The comparison of hospice care education between the Chinese and Japanese medical colleges. Chin J Gerontol.

[46] Zhang Q, Fu J, Ma H (2019). A preliminary study on hospice care for undergraduate doctors and nurses from the perspective of traditional culture. Med Jurisprudence.

[47] Cao WJ, Li CY, Zhang QQ (2022). Perceptions on the current content and pedagogical approaches used in end-of-life care education among undergraduate nursing students: a qualitative, descriptive study. BMC Med Educ.

[48] Beccaro M, Lora AP, Scaccabarozzi G (2013). Survey of Italian general practitioners: knowledge, opinions, and activities of palliative care. J Pain Symptom Manage.

[49] Jung MY, Matthews AK (2021). Understanding nurses' experiences and perceptions of end-of-Life care for cancer patients in Korea: a scoping review. J Palliat Care.

[50] Ismail Y, Shorthose K, Nightingale AK (2015). Trainee experiences of delivering end-of life care in heart failure: key findings of a national survey. Br J Cardiol.

[51] Selman L, Harding R, Beynon T (2007). Improving end-of life care for patients with chronic heart failure: "Let's hope it'll get better, when I know in my heart of hearts it won't". Heart.

[52] Singh GK, Davidson PM, Macdonald PS (2019). The perspectives of health care professionals on providing end of life care and palliative care for patients with chronic heart failure: an integrative review. Heart Lung Circ.

[53] Buss MK, Rock LK, McCarthy EP (2017). Understanding palliative care and hospice: a review for primary care providers. Mayo Clin Proc.

[54] Catt S, Blanchard M, Addington-Hall J (2005). The development of a questionnaire to assess the attitudes of older people to end-of-life issues (AEOLI)[J]. Palliat Med.

[55] Park KS, Yeom HA (2014). Factors influencing Korean nurses’ attitudes towards hospice care. Int Nurs Rev.

[56] Ni K, Gong Y, Li F (2019). Knowledge and attitudes regarding hospice care among outpatients and family members in two hospitals in China. Medicine.

[57] Living Wills Promotion Association (LWPA). Quality End of Life Care for All (QELCA) Train the Trainers. 2022. http://www.lwpa.org.cn/Pub/s/281/3841.shtml. Accessed 19 Sept 2022.

[58] West China Fourth Hospital of Sichuan University. QELCA Course Training Completed Successfully. 2021. https://wc4hospital.scu.edu.cn/info/1175/9302.htm. Accessed 8 Apr 2022.

[59] Living Wills Promotion Association (LWPA). The QELCA Training First Launched in a hospital of Fujian Province. 2022. http://www.lwpa.org.cn/Pub/s/69/5881.shtml. Accessed 25 Sept 2023.

[60] Yang Z, Hou B, Chen P (2021). Preference and influencing factors of advance care planning for Chinese elderly patients with chronic diseases: a mixed-methods approach. J Hospice Palliat Nurs.

[61] Zhang HJ. Make death education become a general education course for college students. Beijing, China: CPPCC DAILY; 2022. p. 11.

[62] Eun YL, Jin YJ, Na YJ (2020). The effects of nurses' knowledge of withdrawal of life-sustaining treatment, death anxiety, perceptions of hospice on their attitudes toward withdrawal of life-sustaining treatment. Hanguk Hosupisu Wanhwa Uiryo Hakhoe Chi.

[63] Jung ML, Joo NJ (2022). Influence of good death perception, moral anguish, and end-of-life care attitude on end-of-life care performance of general hospital health managers. J Environ Public Health.

